# Staphylococcal Enterotoxin C Subtypes Are Differentially Associated with Human Infections and Immunobiological Activities

**DOI:** 10.1128/mSphere.01153-20

**Published:** 2021-01-27

**Authors:** Olivia N. Chuang-Smith, Patrick M. Schlievert

**Affiliations:** a Department of Microbiology and Immunology, University of Minnesota Medical School, Minneapolis, Minnesota, USA; b Department of Microbiology and Immunology, University of Iowa Carver College of Medicine, Iowa City, Iowa, USA; University of Kentucky

**Keywords:** *Staphylococcus aureus*, enterotoxin, superantigen, toxic shock syndrome

## Abstract

Staphylococcus aureus causes significant infections, responsible for toxic shock syndrome (TSS), hemorrhagic pneumonia, and many other infections. S. aureus secretes virulence factors, which include superantigens such as staphylococcal enterotoxins (SEs). We examined differences in immunobiological activities and disease associations among the four human SEC subtypes. We sequenced the *sec* gene from 35 human isolates to determine SEC subtypes. Upon finding differences in disease association, we used a [^3^H]thymidine uptake assay to examine SEC-induced superantigenicity. We also employed a rabbit model of SEC-induced TSS. SEC-2 and SEC-3 were associated with menstrual TSS and vaginal isolates from healthy women, whereas SEC-4 was produced by USA400 isolates causing purpura fulminans and hemorrhagic pneumonia. SEC subtypes differed in potency in a TSS rabbit model and in superantigenicity. There was no difference in superantigenicity when tested on human peripheral blood mononuclear cells. Despite differences, all SECs reacted with polyclonal antibodies raised against the other SEC subtypes. The associations of SEC subtypes with different infections suggest that S. aureus produces virulence factors according to host niches.

**IMPORTANCE** Staphylococcal enterotoxin C has four subtypes that cause human diseases, designated SEC-1 to -4. This study shows that SEC-2 and SEC-3 are the most toxic subtypes in a rabbit model and are associated with human vaginal infections or colonization in association with another superantigen, toxic shock syndrome toxin 1. SEC-4 is associated with purpura fulminans and hemorrhagic pneumonia. SEC-1 is uncommon. The data suggest that there is some selective pressure for the SEC subtypes to be associated with certain human niches.

## Abstract (precis)

Staphylococcal enterotoxin C has four subtypes that cause human diseases, designated SEC-1 to -4. This study shows that SEC-2 and SEC-3 are the most toxic subtypes in a rabbit model and are associated with human vaginal infections or colonization in association with another superantigen, toxic shock syndrome toxin 1.

## INTRODUCTION

Although Staphylococcus aureus can exist as a benign constituent of the human microbiome, it is capable of causing serious diseases such as toxic shock syndrome (TSS), purpura fulminans, and hemorrhagic pneumonia (1–3). The Centers for Disease Control and Prevention previously reported that S. aureus is a highly significant cause of serious infectious diseases in the United States (4). Its numerous secreted exotoxins contribute to the ability of S. aureus to cause pathology in the host. A group of these exotoxins, the superantigens (SAgs), is known to induce massive T-cell proliferation, leading to large amounts of T-cell and macrophage cytokine production (1–3). This resultant “cytokine storm” induces fever, hypotension/shock, and rash, possibly resulting in death. SAg interaction with major histocompatibility complex (MHC) class II molecules on antigen-presenting cells is nonspecific and does not require SAg processing prior to interaction with T lymphocytes through the variable part of the β-chain of the T cell receptor (Vβ-TCR). SAgs stimulate ∼10,000-fold more T lymphocytes than typical antigens (1–3, 5, 6).

One subfamily of SAgs produced by S. aureus is the staphylococcal enterotoxins (SEs), SAgs that are responsible for food poisoning and nonmenstrual TSS, including infections that result secondarily to respiratory viral infections, allergies, and asthma (1, 2). SEs characterized thus far include SEA-SEE and SEG; numerous SE-like SAgs have also been described (1, 2, 7).

**FIG 1:**
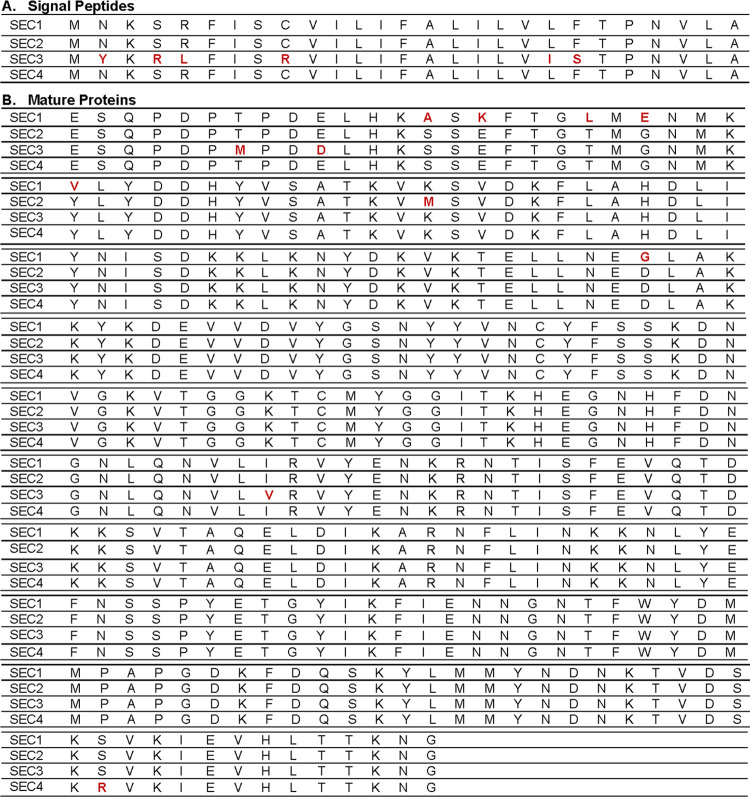
Alignment of SEC subtypes 1 to 4. (A) Alignment of the signal peptide, which is the first 27 amino acid residues of the entire SEC protein, which are cleaved to yield the mature SEC proteins. Differences from the consensus are in red boldface type. Only SEC-3 has residues in the signal peptide that differ from the others. (B) Alignment of the mature SEC proteins. Differences from the consensus are in red boldface type. Most of the differences between the SEC subtype proteins were in the N-terminal region of the mature proteins.

Among the SEs is SEC, which exists as 4 known human subtypes (SEC-1 through SEC-4); they differ from one another by at most 15 amino acids. Most differing amino acids lie in the signal peptide (Fig. 1A) and the N-terminal region of the mature proteins (Fig. 1B). The C-terminal end is more conserved between subtypes and is suggested to be structurally responsible for major biological activities (8). The molecular masses of all SEC subtypes differ only modestly from one another, falling between 24 and 28 kDa (9).

Baba et al. (10) sequenced the community-associated methicillin-resistant S. aureus (CA-MRSA) strain MW2 and noted that its genome carried a new allelic variant of the SEC gene, *sec4*. It has been shown that USA400 strains like MW2, most of which are methicillin resistant, are associated with SEC-4 carriage (11, 12). This led us to hypothesize that the SEC subtypes might each associate with different human infection types based on differences in immunobiological activities. Our current study uses sequencing to determine SEC subtypes carried by S. aureus isolates causing a variety of human diseases. We found associations of certain SEC subtypes with particular diseases, prompting us to ascertain whether there are disparities in SEC subtype immunobiological activities. We observed differences in activities. Currently, the most documented method to neutralize SAgs is intravenous immunoglobulin (IVIG). IVIG was able to neutralize all 4 SEC subtypes.

## RESULTS

### Association of SEC subtypes with S. aureus-induced infections.

Recent studies (10–12) observed that USA400 strains such as MW2 and MNKN, both of which are MRSAs associated with hemorrhagic pneumonia, produce SEC-4. This raises the possibility that SEC-1 to SEC-3 also associate with certain clonal strains of S. aureus and human infection types.

To determine whether such an association is present among isolates, we sequenced the *sec* gene from 35 clinical S. aureus isolates, most of which were from patients with S. aureus infections, with a few isolates from the vaginal flora of healthy women. Interestingly, SEC-1 was the least frequent subtype found among the isolates, observed in only two strains, one responsible for a case of nonmenstrual TSS and one associated with atopic dermatitis (Table 1). The most common subtypes encountered were SEC-2 and SEC-3; the majority of S. aureus isolates producing these SEC subtypes caused menstrual TSS, with coproduction of TSS toxin 1 (TSST-1), or they were from the vaginal flora of healthy patients along with TSST-1. SEC-4 was associated with USA400 S. aureus isolates responsible for purpura fulminans, hemorrhagic pneumonia, and mastitis; all were MRSA. These strains did not produce TSST-1. USA400 strains are likely to cause lethality in such patients (10, 13). We have never observed USA300 strains to produce any SEC subtype, and thus, no USA300 strains were examined in this study.

**TABLE 1:** S. aureus strains used in this study

Patient type(s)	Strain name	Characteristic^*a*^	Reference
Healthy women	29T	USA200 vaginal MSSA	28
	NV22	USA200 vaginal MSSA	28
	NV45	USA200 vaginal MSSA	28
	NV131	USA200 vaginal MSSA	28

Menstrual TSS	CDC587	USA200 vaginal MSSA	29
	MN11	USA200 vaginal MSSA	This study
	MN14	USA200 vaginal MSSA	This study
	MNBC	USA200 vaginal MSSA	This study
	MNDUP	USA200 nasal MSSA	This study
	MNLY	USA200 postinfluenza	This study
	MNLD	USA200 vaginal MSSA	This study
	MNLQ	USA200 vaginal MSSA	This study
	MNPW	USA200 vaginal MSSA	This study
	MNRD	USA200 vaginal MSSA	This study
	MNRJ	USA200 vaginal MSSA	This study
	MNTM	USA200 vaginal MSSA	This study
	MNTNS	USA200 vaginal MSSA	This study
	MNWN	USA200 vaginal MSSA	This study
	MNWZ	USA200 vaginal MSSA	This study

Nonmenstrual TSS	MNCOP	MSSA precursor to USA400 MRSA	30
	MNDON	Heel isolate	31
	MNGN	USA400 MRSA	This study
	MNPE	USA200 MSSA	32

Hemorrhagic pneumonia and purpura fulminans	MW2	USA400 MRSA	33
	MNKN	USA400 MRSA	34
	MNASK	USA400 MRSA	34
	MNNO	USA400 MRSA	This study

Atopic dermatitis	CR39		18
	AD12		18
	AD50		18
	AD53		18
	AD63		18

Food	FRI361	Food poisoning	35
	FRI913	Food poisoning	36

a MSSA, methicillin-sensitive *S. aureus*.

When we compared SEC-2/SEC-3 association with menstrual TSS/healthy-person vaginal isolates to SEC-4 association with nonmenstrual TSS, hemorrhagic pneumonia, and other infections (Table 2), the difference in SEC subtype was statistically significant (*P* < 0.0001). In contrast to the above-mentioned diseases, atopic dermatitis S. aureus isolates had no particular association with any SEC subtype; all subtypes were found among these strains. The overall trends demonstrated that SEC-2 and SEC-3 are associated with vaginal isolates, whether from healthy vaginal microflora or from menstrual TSS patients, where TSST-1 was coproduced, and SEC-4 was associated with MRSA USA400 strains primarily causing serious pulmonary S. aureus infections.

**TABLE 2:** SEC subtypes 1 to 4 associated with human infections

Isolate type(s)	No. of isolates				PFGE designation^*a*^
	SEC-1	SEC-2	SEC-3	SEC-4	
Menstrual TSS/healthy vaginal microflora	0	7	13	0	USA200 TSST-1^+^
Purpura fulminans, hemorrhagic pneumonia, mastitis	1	0	0	9	USA400
Atopic dermatitis	1	2	1	1	Mixed

a PFGE, pulsed-field gel electrophoresis; TSST-1^+^, TSST-1 positive.

### SEC subtypes stimulate rabbit splenocyte proliferation.

**FIG 2:**
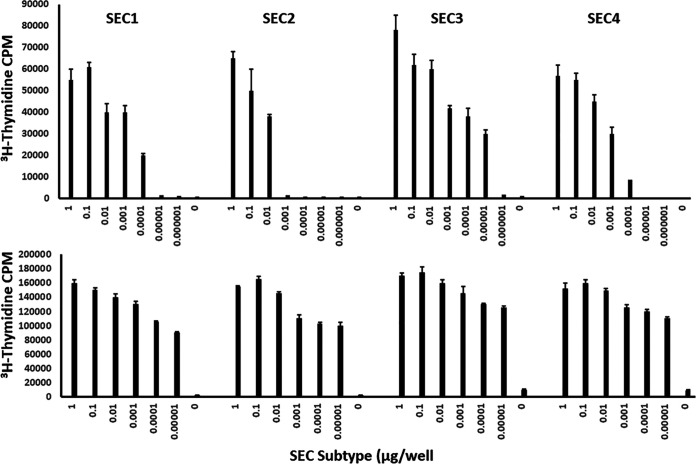
SEC subtypes induce both rabbit and human lymphocyte proliferation. (Top) All SEC subtypes stimulate the proliferation of rabbit splenocytes. Rabbit splenocytes were stimulated with different concentrations of each SEC subtype in a [^3^H]thymidine uptake assay. (Bottom) SEC subtypes 1 to 4 stimulate proliferation of human lymphocytes. Human PBMCs were stimulated with differing concentrations of SEC toxins in a [^3^H]thymidine uptake assay.

Because we observed that certain SEC subtypes were associated with different S. aureus infections, we examined whether there might be differences in the immunobiological properties of the SEC subtypes. One possible difference between the SEC subtypes would be in superantigenicity, or the ability to stimulate T-cell proliferation. In a [^3^H]thymidine uptake assay with rabbit splenocytes, we observed that all SEC subtype toxins were able to promote lymphocyte proliferation in a dose-dependent manner (Fig. 2A). However, proliferation levels differed among the 4 subtypes; SEC-2 stimulated the lowest levels of lymphocyte proliferation at most concentrations of toxin. At 0.001 μg/well, SEC-2-induced proliferation dropped to background levels, whereas this decrease was not observed for other SEC subtype toxins until 0.00001 μg/well. Strikingly, SEC-3 toxin stimulated the highest proliferation levels, remaining at 15-fold-higher levels at 0.00001 μg/well, when other SEC subtypes stimulated only background levels of lymphocyte proliferation. Although we observed differences in splenocyte proliferation levels induced by each SEC subtype, all SEC subtypes demonstrated superantigenicity.

### SEC subtypes 1 to 4 stimulate proliferation of human lymphocytes.

Since we observed that SEC-1 to -4 induced rabbit splenocyte proliferation and that differences were present among the proliferation levels stimulated, we investigated the effect of the SAgs on human lymphocytes. The [^3^H]thymidine uptake assay performed with peripheral blood mononuclear cells (PBMCs) demonstrated that the SEC subtypes were all able to generate proliferative responses, also in dose-dependent manners (Fig. 2B). However, the attenuated superantigenicity of SEC-2 seen with rabbit splenocytes was not observed with human PBMCs. The increased superantigenicity of SEC-3 in comparison to the other subtypes was less apparent in the human PBMC assay, but at all concentrations of SEC, SEC-3-induced PBMC proliferation was slightly higher than those of the other subtypes. Thus, we found that all SEC subtypes induce proliferative responses in human PBMCs, but the differences in superantigenicities were not as apparent as the rabbit splenocyte response to SEC subtypes.

**FIG 3:**
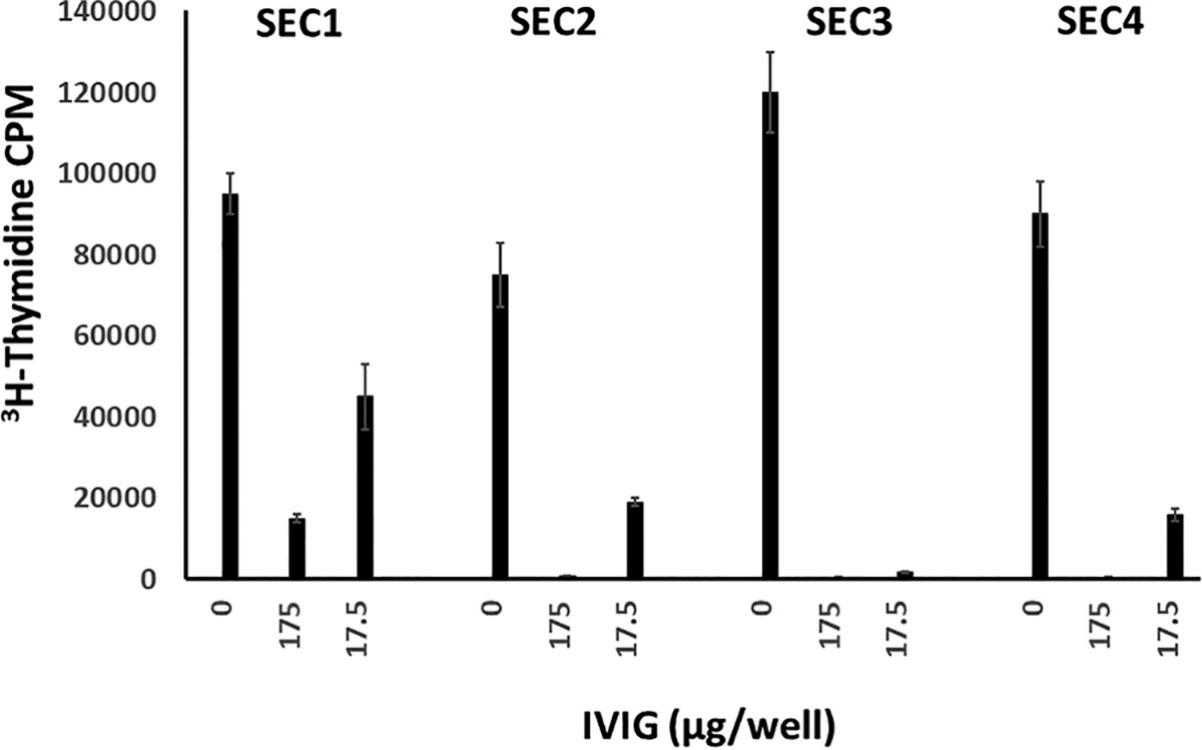
IVIG neutralizes the activity of all four SEC subtypes. Human PBMCs were incubated in the presence of 0.01 μg/well SEC and differing concentrations of IVIG. Bars indicate levels of lymphocyte proliferation as the incorporation of [^3^H]thymidine into DNA in counts per minute (CPM). Both IVIG concentrations neutralized superantigenicity with a *P* value of <0.01 as determined by Student’s *t* test.

We treated PBMCs with IVIG along with SECs, as IVIG can also neutralize S. aureus SAgs (14). IVIG was effective in neutralizing SEC-induced lymphocyte proliferation (Fig. 3). There were small differences in IVIG neutralization abilities, which may reflect small epitope differences among the SECs, which are known to exist.

### SEC-3 demonstrates the greatest lethality in a TSS rabbit model.

To continue elucidating the basis for SEC subtype disease associations (Table 3), we examined the ability of each SEC subtype to enhance lipopolysaccharide (LPS)-induced lethality in Dutch-belted rabbits. Staphylococcal SAgs are known to synergize with LPS, causing fever, diarrhea, and lethal shock (15). When Dutch-belted rabbits were administered 0.001, 0.01, 0.1, and 10 μg/kg of body weight of SEC with the subsequent injection of 10 μg/kg of LPS, the most lethal SAgs were SEC-3, which yielded the lowest 50% lethal dose (LD_50_), at 0.001 μg/kg (Table 3), and SEC-2, which exhibited an LD_50_ of 0.002 μg/kg, while the LD_50_s for SEC-1 and SEC-4 were 0.01 μg/kg.

**TABLE 3:** LD_50_s of SEC subtypes in a rabbit model of TSS

SEC subtype administered	LD_50_ (μg/kg)
SEC-1	0.01
SEC-2	0.002
SEC-3	0.001
SEC-4	0.01

### Structural studies of SEC subtypes.

**FIG 4:**
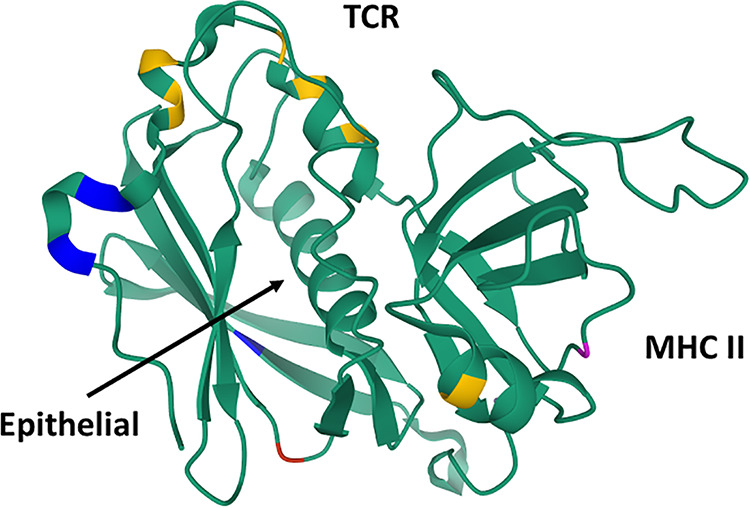
Protein Data Bank three-dimensional (3-D) ribbon model of SEC-2 (accession number 1STE) showing amino acid variations (colored residues). Yellow, amino acid differences from the consensus in SEC-1; magenta, difference from the consensus in SEC-2; blue, differences from the consensus in SEC-3; red, difference from the consensus in SEC-4. The MHC class II-binding domain on SECs includes multiple amino acids on the right side (in the standard view) of the molecules. The Vβ-TCR-binding sites are in a groove at the top of the SECs. The epithelial cell-binding site (CD40) encompasses the central diagonal α-helix with surface amino acids exposed on the back side of the SECs.

In preliminary studies to hypothesize the reason for differences in the biological activities of SEC subtypes, we mapped the locations of the amino acid differences to assess if these changes are in regions of known biological activities. Figure 1A and B show the location of the amino acid variations in the SEC-1 to -4 subtypes. Figure 4 shows a cartoon protein ribbon image of the structural locations of these variations based on data in the RCSB Protein Data Bank (structure of staphylococcal enterotoxin C2 under accession number 1STE). There are no variations in amino acids in the central diagonal α-helix that appears to control the interaction with CD40 on epithelial cells (16). SEC-2 has one amino acid difference, which is located in the MHC class II-binding region (Lys39 to Met) (17). It is possible that the increased toxicity of this SEC variant is the result of increased MHC class II binding. The SEC-3 subtype has multiple amino acid differences, and the majority of the differences are in the general location of the Vβ-TCR-binding site (17). It is again presumed that these changes in amino acids increase the interaction with the TCR. Such amino acid differences could account for the increased biological activities of these two SEC subtypes. SEC-1 and -4 have reduced toxicity, and changes are located in regions not shown to affect activity. SEC-1 and -4 thus may have baseline SEC toxicity. We have not attempted mutagenesis of these amino acids individually because of the large number of individual changes needed and multiple LD_50_s required in rabbits.

## DISCUSSION

In this study, we sought to dissect possible differences among the four human-associated SEC subtypes, examining their relationships to human infections and their ability to stimulate the immune system. In addition to finding no additional human SEC subtypes other than the already described four subtypes, we found two groups of SEC subtype associations with human illnesses: (i) SEC-2/SEC-3 association with menstrual TSS and vaginal isolates from expectedly healthy women and (ii) SEC-4 association with USA400 strains responsible for primarily serious pulmonary illnesses, including purpura fulminans and necrotizing pneumonia. Interestingly, atopic dermatitis S. aureus isolates had no association with any single SEC subtype; all SEC subtypes were found among these strains. These data suggest that persons with damaged skin, as in atopic dermatitis, are susceptible to colonization/infection with nearly any type of S. aureus strain. These skin isolates likely serve as reservoirs that give rise to a myriad of other infections (18, 19). The same has been proposed for mucosal isolates, but the unique association of vaginal mucosal isolates with SEC-2/SEC-3 suggests that these isolates are more restricted in the host niche; the isolates in our study also coproduced TSST-1 that was the cause of menstrual vaginal-associated TSS.

During a skin isolate’s transition to a mucosal surface (i.e., the vaginal surface), transcriptional regulatory mechanisms could alter the expression profile of toxins, surface proteins, and other virulence factors to better adapt the strain to its new environment (19). We know that nearly all menstrual TSS S. aureus isolates are positive for TSST-1. TSST-1 is capable of easily crossing epithelial barriers, whereas SEs are not as able to do so (20). However, coproduction of TSST-1 with SEC-2 or SEC-3 may facilitate SEC penetration of mucosal surfaces. To cause systemic illness in the vaginal environment, the toxin must cross the epithelial barrier. Most atopic dermatitis isolates in this study coproduced TSST-1, and thus, this SAg’s expression could be upregulated once the skin isolate reaches the vaginal mucosa, enabling the strain to cause TSS.

At least two possibilities can explain the disease association with certain SEC subtypes. It is possible that unique activities of some SEC subtypes allow them to facilitate certain infection types. Alternatively, different SEC subtypes could be markers for some other property responsible for the association. The SEC-2 and SEC-3 association with menstrual TSS and vaginal isolates from healthy women indicates that these SEC subtypes might synergize with TSST-1 during TSS or simply accompany strains that produce TSST-1. SEC does not cross epithelial barriers readily, but TSST-1 has this ability when these toxins are administered vaginally in a rabbit model (20). Thus, it is possible that as TSST-1 opens the vaginal mucosal barrier to cause menstrual TSS, SEC-2 or SEC-3 could facilitate disease causation. In our unpublished studies, we have shown that similar doses of multiple superantigens are more toxic to rabbits than single SAgs. Additionally, our laboratory has shown that alpha-toxin can inflict damage on the vaginal epithelial barrier by direct cytotoxicity and inducing localized inflammation, allowing for enhanced penetration of TSST-1 (21) and, thus, possibly of SEC-2 and SEC-3. In the current study, we also observed that when SEC-2 and SEC-3 were administered intravenously (i.v.) to Dutch-belted rabbits, these two subtypes yielded the lowest LD_50_s in the presence of LPS. The enhanced virulence of SEC-2 and SEC-3 seen in this model could support their association with menstrual TSS. One way for S. aureus to inactivate the immune system through SAg production is to select for SAgs with greater activity such that even small amounts may have large effects on the host. We also found that SEC-2 and SEC-3 were associated with vaginal isolates of healthy women; the presence of these isolates in the vaginal microbiome is consistent with these isolates giving rise to menstrual TSS.

As for the SEC-4 association with USA400 isolates, our findings confirm results from Shukla et al. (11). USA400 strains are particularly known for their lethality, and in the study by Baba et al. (10), CA-MRSA USA400 strain MW2 was sequenced to determine genetic reasons for this strain’s increased lethality. Several new virulence genes were found in the genome, including *sec4*. In the current study, SEC-4 was not the most toxic in activity compared to other SEC subtypes. It stimulated levels of human lymphocyte proliferation similar to those of SEC-1, whereas with rabbit splenocytes, SEC-4 was not as potent as SEC-1. Similarly, the LD_50_ for SEC-4 and SEC-1 was 0.01 μg/kg in our rabbit model, implying that these SEC subtypes are not as lethal as SEC-2 and SEC-3 in causing TSS, at least in that model. These results suggest that SEC-4 is likely to contribute importantly to human necrotizing pneumonia, as demonstrated by Strandberg et al. (22), but also serves as a marker for USA400 strains. In those necrotizing pneumonia studies, histological evidence revealed hemorrhage and the absence of air spaces in lung tissue when either MW2 (USA400 strain) or purified SEC-4 was administered intratracheally to rabbits. Immunization with SEC-4 prevented the occurrence of respiratory distress and lethality.

Our studies also demonstrated differences among subtypes in superantigenicity as tested with rabbit splenocytes and human PBMCs. All 4 SEC subtypes were superantigenic. In rabbits, SEC-2 was less active than the other 3 SEC subtypes, and SEC-3 was more active in both rabbit and human systems. These differences in superantigenicity do not appear to result from direct amino acid changes altering interactions with TCRs and MHC class II molecules since the observed changes are outside the contact areas. Instead, the amino acid changes likely alter superantigen stability or cause minor structural alterations that result in activity differences.

Finally, IVIG was successful in neutralizing SEC-induced lymphocyte proliferation and has been used in treating SAg-related S. aureus infections (14). It has become a less expensive treatment to neutralize SEC than a few years ago.

## MATERIALS AND METHODS

### SEC subtype determination in clinical S. aureus isolates.

S. aureus isolates were grown in Todd-Hewitt (TH) broth until stationary phase. Cultures were pelleted (14,000 × *g* for 5 min) and resuspended in lysis buffer (20 mM Tris–10 mM EDTA, 2.7 mg/ml lysozyme, 1.4 mg/ml lysostaphin, 1 μl per 91 μl Triton X-100). Using the Qiagen (Valencia, CA) DNeasy blood and tissue kit, genomic DNA was purified from cell lysates. The *sec* gene from each isolate’s DNA was amplified by PCR with *sec*-specific primers (forward primers GCGTAATTTTGATATTCGCACTTATA, CAAGATGCTTAGAAATCCTCTG, and CCTTGAGAAAGAGTTTTGTATATAAG and reverse primers TTATCCATTCTTTGTTGTAAGGTG, TCACTGTATAAATAACCGCACTTTC, and TTAGATTCACTGTATAAATAACCGCACTTTC). PCR-amplified DNA was sequenced by the University of Minnesota Biomedical Genomics Center.

### SEC toxin purification.

As described previously (20), cultures of S. aureus strains MNDON, FRI361, FRI913, and MW2 (strains expressing SEC-1, -2, -3, and -4, respectively) (Table 1) were grown overnight aerobically in 25 ml of TH broth at 37°C. Cultures were individually diluted 1:120 into dialyzable beef heart medium supplemented with 1% glucose-phosphate buffer, with growth at 37°C until stationary phase (23). Exoproteins in cultures were precipitated with an 80% final concentration of ethanol, and precipitates were collected by centrifugation, resuspended in 75 ml pyrogen-free water, and dialyzed overnight against pyrogen-free water. The dialyzed toxin solution was subjected to isoelectric focusing twice, in pH gradients of 3.5 to 10. Double immunodiffusion was utilized to identify SEC-containing fractions; reactive fractions were pooled and dialyzed against pyrogen-free water. Pooled fractions were electrophoresed on SDS-PAGE gels to verify purity, and toxins were stored lyophilized until used.

### Isolation of rabbit splenocytes and human PBMCs.

The spleen from a New Zealand White rabbit was removed aseptically and placed into RPMI 1640 medium (Lonza, Walkersville, MD) supplemented with 2% fetal bovine serum, 1% penicillin-streptomycin, and 200 μM glutamine (24). Cell suspensions were obtained by teasing into RPMI 1640 medium, with cell washing in RPMI 1640 medium. Spleen cells were resuspended in RPMI 1640 medium to obtain a concentration of 1.0 × 10^6^ cells/ml and aliquoted (200 μl/well) into a 96-well flat-bottom culture plate (Becton, Dickinson, Franklin Lakes, NJ) for lymphocyte proliferation assays.

Heparinized (10 U/ml) human blood was obtained in compliance with an approved University of Minnesota IRB protocol (1004M80313). Blood was layered onto a Histopaque-1077 gradient solution (Sigma, St. Louis, MO), and cells at the interface (PBMCs) were collected and washed with RPMI 1640 medium (6). PBMCs were suspended in a volume of RPMI 1640 medium for a final concentration of between 5.0 × 10^5^ and 1.0 × 10^6^ cells/ml.

### Rabbit and human lymphocyte proliferation assays.

Purified rabbit and human lymphocytes were incubated with different concentrations of SEC subtypes (0.1 pg to 1 μg per well) for 3 days at 37°C in 5% CO_2_, as described previously (6). For some experiments, we used IVIG (1.74, 17.4, and 174.0 μg/well) to neutralize SEC subtypes, as it has been shown to neutralize S. aureus SAg activity (14). After 3 days, 1 μCi of [^3^H]thymidine was added to each well of lymphocytes, with exposure for an additional 24 h. A Mash II apparatus (Microbiological Associates, Bethesda, MD) was used to collect cellular DNA onto glass fiber filters; thymidine uptake was measured using a liquid scintillation counter (model LS; Beckman Instruments, Fullerton, CA). Data were expressed as averages from four replicates, in counts per minute. Experiments testing the effects of different SEC subtype concentrations on PBMCs were repeated at least 4 times; plots shown represent total data.

### SEC-induced enhancement of lipopolysaccharide shock in Dutch-belted rabbits.

A rapidly fatal model of TSS in rabbits can be tested based on the ability of SAg to amplify the lethal effects of lipopolysaccharide (LPS) through synergistic tumor necrosis factor alpha (TNF-α) production. We used this model to compare the lethal properties of all 4 SEC subtypes. Young adult rabbits were administered 0.001, 0.01, 0.1, or 10.0 μg/kg of SEC toxin (SEC-1, SEC-2, SEC-3, or SEC-4) dissolved in pyrogen-free phosphate-buffered saline (PBS) intravenously via the marginal ear vein, as described previously (25, 26). At least three rabbits were administered each SEC dose. Four hours after the administration of SEC subtypes, 10 μg/kg of LPS from Salmonella enterica serovar Typhimurium was given intravenously. Rabbits were monitored for lethality and euthanized with phenytoin-pentobarbital (Beuthanasia D; Schering-Plough Animal Health Corporation, Union, NJ) (1 ml/kg) if they exhibited failure to right themselves and escape behavior. All experiments were conducted according to the guidelines of the University of Minnesota Institutional Animal Care and Use Committee. The lethal dose 50% endpoint (LD_50_) values were calculated according to the technique of Reed and Muench (27).

### Statistical analysis.

Fisher’s exact test was utilized to calculate the statistical significance of SEC subtype associations with human illness. Student’s *t* test was used to assess differences in the results of lymphocyte proliferation assays.
